# Classification and localization of maize leaf spot disease based on weakly supervised learning

**DOI:** 10.3389/fpls.2023.1128399

**Published:** 2023-05-08

**Authors:** Shuai Yang, Ziyao Xing, Hengbin Wang, Xiang Gao, Xinrui Dong, Yu Yao, Runda Zhang, Xiaodong Zhang, Shaoming Li, Yuanyuan Zhao, Zhe Liu

**Affiliations:** ^1^ College of Land Science and Technology, China Agricultural University, Beijing, China; ^2^ Key Laboratory of Remote Sensing for Agri-Hazards, Ministry of Agriculture and Rural Affairs, Beijing, China

**Keywords:** deep learning, crop diseases, interpretable AI, image classification, weakly supervised learning

## Abstract

Precisely discerning disease types and vulnerable areas is crucial in implementing effective monitoring of crop production. This forms the basis for generating targeted plant protection recommendations and automatic, precise applications. In this study, we constructed a dataset comprising six types of field maize leaf images and developed a framework for classifying and localizing maize leaf diseases. Our approach involved integrating lightweight convolutional neural networks with interpretable AI algorithms, which resulted in high classification accuracy and fast detection speeds. To evaluate the performance of our framework, we tested the mean Intersection over Union (mIoU) of localized disease spot coverage and actual disease spot coverage when relying solely on image-level annotations. The results showed that our framework achieved a mIoU of up to 55.302%, indicating the feasibility of using weakly supervised semantic segmentation based on class activation mapping techniques for identifying disease spots in crop disease detection. This approach, which combines deep learning models with visualization techniques, improves the interpretability of the deep learning models and achieves successful localization of infected areas of maize leaves through weakly supervised learning. The framework allows for smart monitoring of crop diseases and plant protection operations using mobile phones, smart farm machines, and other devices. Furthermore, it offers a reference for deep learning research on crop diseases.

## 1 Introduction

Maize, a crucial food and industrial crop, is vulnerable to various diseases, including corn brown spot, corn southern leaf blight and common rust. These diseases can have a significant impact on the quality and yield of maize during its growth (Rosenzweig et al., 2001). Crop production monitoring involves the identification of disease types and the localization of susceptible areas. This is essential in creating plant protection prescriptions and ensuring automatic and precise applications. Currently, the detection and identification of maize diseases through artificial intelligence techniques is a hot topic of research. The use of this technology can play a significant role in accurately identifying disease types and their location, therefore improving crop production monitoring.

Automated disease identification, made possible by a computerized control system, allows for early detection and monitoring of diseases. This system provides a reference for agricultural workers, which helps to manage and mitigate the damage caused by these diseases in a timely manner (Singh et al., 2020). Initially, researchers commonly employed machine learning (ML) models, specifically support vector machines (SVM) (Elangovan and Nalini, 2017), decision trees (DT) (Rokach and Maimon, 2005), random forests (RF) (Ramesh et al., 2018), and k-nearest neighbors (KNN) (Liao and Vemuri, 2002), to detect and classify crop diseases. However, the increasing prevalence of deep learning (DL) technology (Shivam and Kumar ,; Kohli et al., 2022) contributed to its utilization within the field of agriculture. DL has proven to be highly versatile and has been adeptly utilized to achieve significant breakthroughs in the realm of agriculture.

Among the deep learning tools, the most commonly used is the convolutional neural network (CNN) (Verma et al., 2021), which requires fewer artificial neurons than traditional feed-forward neural networks, has good performance in classification and recognition, and is widely used in disease identification. For example, Singh et al (Singh et al., 2015) demonstrated the application of an SVM classifier to differentiate between healthy and diseased rice plants, achieving an accuracy of 82%. However, ML-based crop disease identification systems have limitations despite their ability to classify with small amounts of training data. This is because these systems depend heavily on pre-processing and feature selection methods that rely heavily on the agricultural knowledge of experts. Furthermore, selecting a large number of features leads to computational difficulties, while a smaller feature set results in suboptimal classification. Thus, the performance of ML-based crop disease identification systems is inherently limited. Dwivedi et al. introduced a framework, region-based CNN (RCNN), for locating and classifying grapevine plant diseases. Initially, ResNet18 was used to compute depth features that were later classified by the RCNN classifier and produced an improved result of 99.93% for classifying several diseases of grapevine plants, with the disadvantage of poor performance in the real world (Dwivedi et al., 2021). Akshai et al. introduced several DL-based frameworks, namely VGG, ResNet, and DenseNet methods, for computing deep features and classifying plant diseases from the input samples. The method obtained the best performance of the DenseNet framework with 98.27% accuracy, but at the cost of increased computational complexity (Akshai and Anitha, 2021). Batool et al. proposed a method to locate several tomato leaf-influenced regions. The AlexNet model was applied to compute the depth features from the input image for training the KNN classifier. The results proved that this method had an accuracy of 76.1%. However, the KNN method is a tedious and time-consuming algorithm (Batool et al., 2020). Although good progress has been made in all of the above studies, in practical applications, given the equipment and time constraints, on top of first ensuring the generalization robustness and recognition accuracy of the disease classifier. We need efficient models with less computational effort and faster inference (Yang et al., 2023). To our knowledge, few studies have explored the applicability of state-of-the-art lightweight deep-learning classification models in the field of maize leaf disease identification.

In terms of data acquisition, most of the images used in current studies are captured in a controlled environment (Subramanian et al., 2022), with monotonous backgrounds and images acquired in a destructive manner. For example, when Mohanty et al. applied a model trained using the PlantVillage database to images from an online resource, the accuracy rate quickly dropped to below 50% (Mohanty et al., 2016). Thus, when DL classifiers are used in natural environments, there are many uncontrollable factors that affect them. The potential use of these studies is limited by the environment in which they are taken, which is why most results in many of the relevant studies show high accuracy, while recognition accuracy decreases when faced with truly complex natural environments. Existing open source disease datasets, such as PlantVillage and CVPR 2020-FGVC7, are not appropriate for real field environments. Thus, there is a need to create a dataset for detecting maize leaf disease in a natural field setting.

Benefiting from the advanced CNN architecture, fully supervised semantic segmentation methods have achieved remarkable performance (Gao et al., 2019). However, these segmentation methods rely heavily on large-scale training samples with pixel-level annotations. Building pixel-level accurate segmentation datasets is very expensive (Jiang et al., 2021a). On the other hand, deep learning classifiers lack interpretability, giving good results but without any explanation or details about the classification mechanism. Especially for crop disease classification, the user also needs to know how these classification results are achieved and what symptoms the disease has. The use of visualization techniques to explore the working mechanisms of deep learning has become a hot topic of research in recent years. Due to the accessibility of image-level labels and the desire to save time and manpower, weakly supervised learning is utilized to achieve semantic segmentation. By employing weakly supervised learning algorithms, classifier designers can enhance their classifiers’ performance. Meanwhile, visualization algorithms can aid users in identifying symptoms and infected areas to achieve a better comprehension of plant diseases. DeChant et al. developed a novel approach for quantifying the likelihood of specific disease types in plants, which involved utilizing various combinations of convolutional neural networks (CNNs) and generating heatmaps as input to images of diseased plants. This methodology offers a valuable tool for non-specialist farmers who can learn about plant diseases *via* deep learning classifier visualizations. Additionally, classifier designers and agricultural experts can study the behavior of classifiers through visualizations generated by the CNNs (Dechant et al., 2017). Brahimi et al. developed a saliency map to visualize plant disease symptoms and identified 13 types of plant diseases. They used multiple filters to pinpoint the location of disease spots (Brahimi et al., 2018). Lu et al. employed a CNN to detect disease spots on rice plants by generating feature maps (Lu et al., 2017). Mohanty et al. compared the performance of AlexNet and GoogleNet by using the publicly available PlantVillage dataset and evaluating performance metrics in 3 scenes (color, greyscale, and segmented) and using visual activation to display disease patches (Mohanty et al., 2016). Therefore, how to make use of the rich semantic information contained in the image-level annotated data and to achieve a weakly supervised semantic segmentation that is close to the semantic segmentation effect by relying only on image-level annotation is a hot topic of research in recent years in the direction related to semantic segmentation, and there are few studies to quantitatively evaluate the applicability of weakly supervised learning in the field of crop disease detection.

To address the issues arising from the above study, we constructed a dataset of field images of maize leaf spot disease for use in natural environments. The deep learning classification models trained on this dataset are more suitable for the identification and detection of maize leaf spot disease in the field. A combination of four lightweight networks and four advanced CAM methods were selected to test the performance of the networks and CAM on the validation set. Also, migration learning was employed to enhance the learning ability of the models during their training. The main contributions of this study can be summarized as follows:

1. We constructed a maize leaf spot field image dataset by combining a smartphone captured maize leaf spot image dataset from maize growing areas in Dezhou City, Shandong Province, and Hebi City, Henan Province, and some open source maize leaf part image datasets. A total of 9401 images were included, and with the help of experts in related fields, image-level annotation was applied to these images, which included five types of maize leaf spot disease (large spot, small spot, curvular leaf spot, brown spot and rust) and one healthy type.
2. We investigated the applicability of four state-of-the-art lightweight networks in the field of crop disease detection and proposed a method combining a disease classifier with CAM visualization for localizing infected areas on maize leaves relying only on image-level annotation. A comprehensive evaluation of the ability of four advanced CAM methods (LayerCAM, XGradCAM, AblationCAM, ScoreCAM) to localize infected areas of disease spots is also presented.
3. We investigated whether class activation mapping maps can be used for disease localization in the field of plant pests and diseases. Analysis of the mIoU of different classification models under different conditions (different combinations of CAM methods and different operational thresholds for generating image boundaries from the score map). our study shows the feasibility of coarse localization of infected areas in maize leaves by means of a combination of lightweight classification models and CAM.

The remainder of the paper is organized as follows: in Section 2, the process of data collection and enhancement is described. Section 3 describes the methods used to classify and localize maize leaf spot disease and the performance evaluation metrics. Experimental details and results are given in section 4. Section 5 presents the conclusions drawn from this study.

## 2 Dataset

### 2.1 A dataset of maize foliar diseases collected from the field’s natural environment

**Figure 1:**
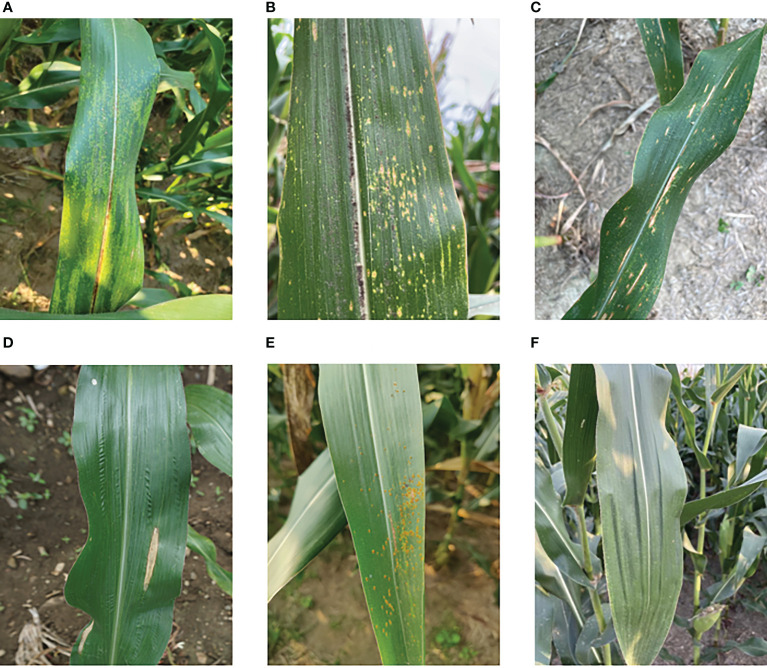
Data set of maize foliar diseases: **(A)** corn brown spot, **(B)** maize curvularia leaf spot, **(C)** corn southern leaf blight, **(D)** corn northern leaf blight, **(E)** common rust, **(F)** healthy.

Data for this experiment were collected in a non-destructive manner *via* iPhone 12 and Samsung Galaxy S21 at experimental fields in Hebi, Henan Province, and Dezhou, Shandong Province. The collection time was mid to late September 2022 and in addition, these maize diseases were all naturally occurring. When photographing, different angles and distances are used, and it is important to ensure that the images cover as many complex backgrounds as possible, such as sky, soil, and weeds. Healthy maize leaves were obtained from the publicly available maize dataset on the Kaggle website and the maize maculates dataset was provided by T Wiesner-Hanks (Wiesner-Hanks et al., 2018), both of which were screened and collated to form the maize leaf disease dataset to be used in this study. **Figure 1** displays six types: 1474 corn brown spot, 1242 corn southern leaf blight, 2407 maize curvularia leaf spot, 615 common rust, 1253 corn northern leaf blight, and 2410 healthy types.

### 2.2 Image pre-processing

Deep learning often relies on large datasets, but in the real world, it is very difficult to collect training data and requires experts in the field to perform the labeling task. In addition, class imbalance and insufficient amount of data are key factors that lead to poor recognition (Öztürk et al., 2021). To address this issue, in this study we use traditional data augmentation to expand the number of samples. The data augmentation mainly involves flipping, color changing, panning, rotating, and resampling the training samples, which is done to enhance the generalization ability of the model and prevent overfitting. First we divide the data into a training set and a validation set in the ratio of 7:3, and then augmented the training set with data. The details are shown in **Table 1**.

**Table 1:** Enhanced dataset.

Disease category	Original	Train	Augmented train	Val
Corn Brown Spot	1474	1032	2064	442
Corn Southern Leaf Blight	1242	870	2040	372
Maize Curvularia Leaf Spot	2407	1685	2066	722
Common Rust	615	431	2155	184
Corn Northern Leaf Blight	1253	878	1998	375
Healthy	2410	1687	2097	723
Total	9401	6583	12420	2818

## 3 Methods

### 3.1 Classification model of maize leaf disease

The well-established CNN architectures in computer vision, such as AlexNet, GoogleNet, VGGNet, and ResNet, are the most popular deep-learning models in image recognition. And they are widely used in the field of plant disease classification. Although they have a good performance in image classification, they also generally have the problem of high memory requirements and high computing power. This makes them almost unusable in some remote areas where internet speeds are very slow. There is therefore a need for lightweight and relatively high-accuracy networks that can be deployed to run on mobile and embedded devices to identify maize leaf spots. In CNN models, there is a trade-off between classification accuracy and model size, and the advent of lightweight networks has greatly improved the efficiency and accuracy of the models. In this study, we selected four advanced lightweight CNNs for this study, and provide a description of each one below.

#### 3.1.1 MobileNetV3

**Figure 2:**
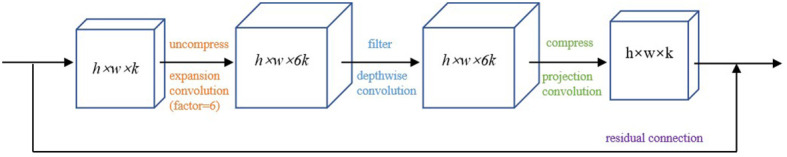
Inverted Residual Linear Bottleneck.

**Figure 3:**
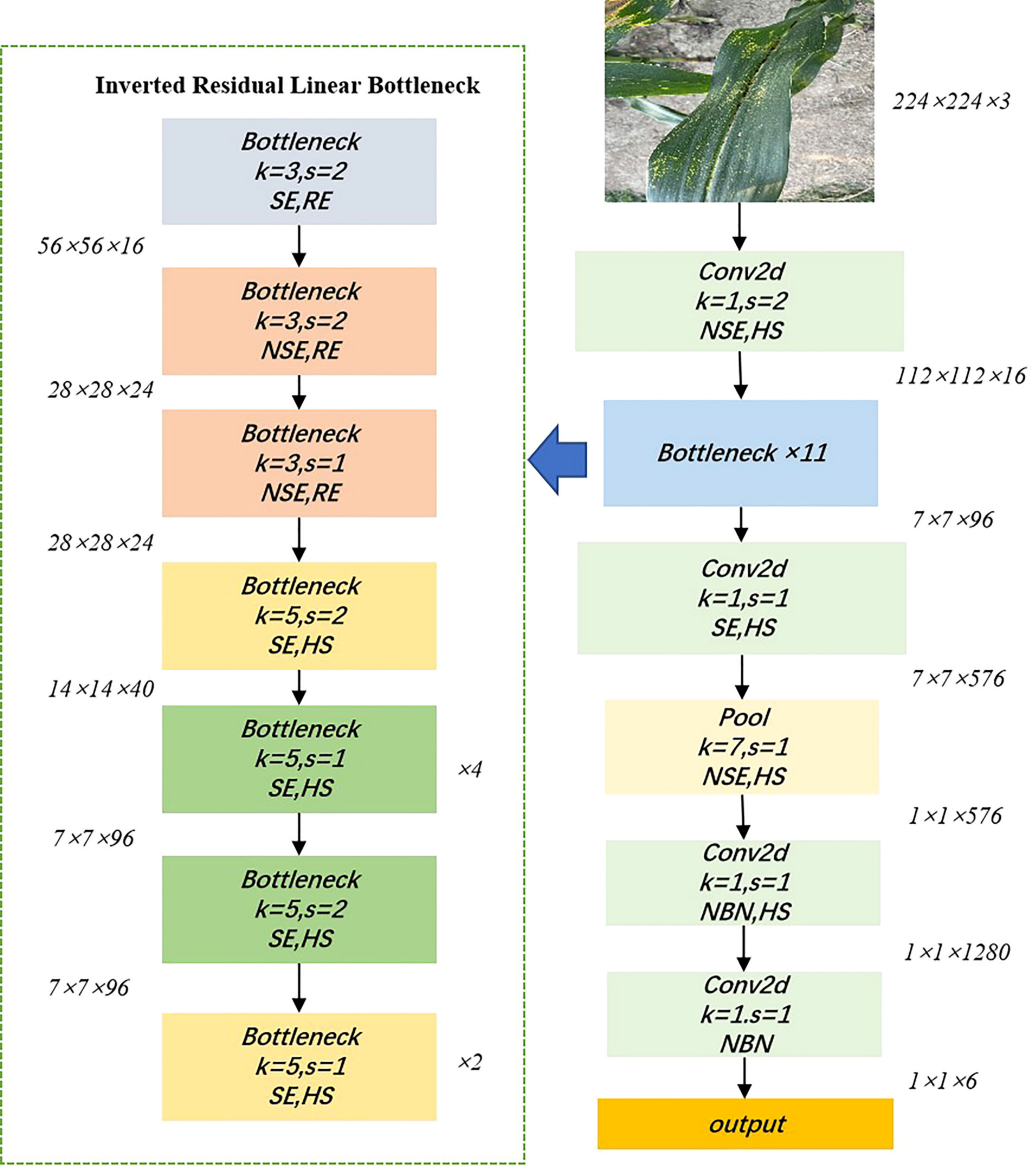
Network structure diagram of MobileNetV3_small. NSE and SE indicate the presence of squeeze and excite layers in the block, HS indicates that the activation function is Hard-Swish, RE indicates that the activation function is ReLU, s indicates the step size, k indicates the convolutional kernel size, and NBN indicates that there is no BN layer.

MobileNetV3 was issued in 2019 and proposed by the Google team (Howard et al., 2019), with excellent performance and speed thanks to the accumulation of the first two generations of V1 and V2. MobileNetV1 consists of a stack of depth-separable convolution modules, which is a decomposition of the standard convolution into a depthwise convolution and a 1×1 pointwise convolution, greatly reducing the computational effort of the network (Howard et al., 2017). MobileNetV2 introduces a linear bottleneck and reverse residual structure (shown in **Figure 2**) in order to produce a more efficient layer structure by exploiting the low-rank nature of the problem (Sandler et al., 2018). When the inputs and outputs have the same number of channels, the inputs and outputs are connected to the residual connections. This structure is extended internally to higher dimensional feature spaces to increase the expressiveness of the non-linear multi-channel transform. The main improvements of MobileNetV3 are as follows: 1) inherited the deeply separable convolution of V1. 2) inherited the residual structure with linear bottlenecks of V2. 3) introduced the SE channel attention structure. 4) used the NetAdapt algorithm to obtain the optimal number of convolution kernels and channels. 5) used a new activation function Hard-Swish instead of ReLu6. In summary, MobileNetV3 incorporates the structures of the previous two and uses NAS (Neural Architecture Search) to search for the configuration and parameters of the network. Two versions are available, which can be defined as MobileNetV3_small and MobileNetV3_large, with different architectural complexity, depending on the demand for resources. **Figure 3** shows the network structure of MobileNetV3_small.

#### 3.1.2 ShuffleNetV2

**Figure 4:**
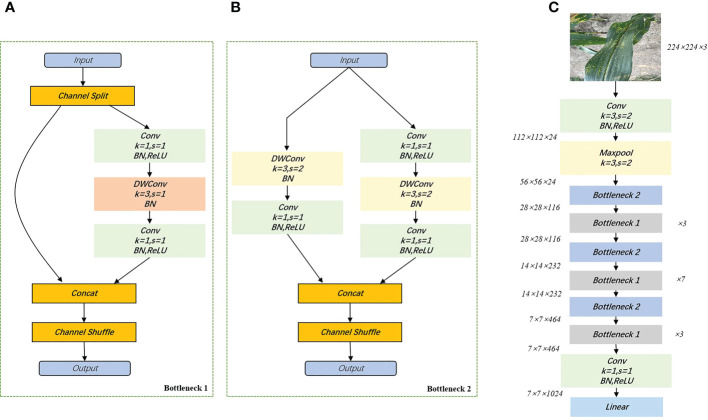
Building blocks of ShuffleNetV2. **(A)**: basic cells; **(B)**: cells used for spatial downsampling; **(C)**: network structure diagram of ShuffleNetV2_1.0×. DWConv, depthwise convolution.

ShuffleNetV2 is a new lightweight neural network proposed in 2018 (Ma et al., 2018), which is an upgraded version of ShuffleNetV1 based on channel shuffling and four efficient network design criteria (G1. Same channel width minimizes MAC. G2. Too much group convolution increases MAC. G3. Internal network fragmentation operations reduce parallelism. G4. Element-wise operations cannot be ignored). Accuracy outperforms other lightweight models at the same complexity. As shown in **Figure 4**, ShuffleNetV2 divides the inputs of the feature channels into two branches, one of which reduces network fragmentation and increases parallel efficiency. The other branch consists of three convolutions with the same input and output channels. It allows each convolutional kernel to run only on the corresponding channel grouping, which minimizes memory access cost (MAC). The advantages of ShuffleNetV2 are: 1) it is efficient in each building block, thus utilizing more feature maps and a larger network capacity. 2) feature reuse, because of channel splitting, so that half of the features are passed directly to the next module. The feature reuse information decays exponentially with the distance between the two modules. That is, the number of feature maps in layer i+j containing layer i feature maps is $r^{j}c$ , where c is the number of feature maps in layer i and r is the parameter of channel splitting. ShuffleNetV2 can set the channel of each basic unit, e.g. 0.5×, 1×, 1.5×, and thus adjust the complexity of the model. For this study, a version of ShuffleNetV2 with 1.0× output channels was chosen.

#### 3.1.3 EfficientNet

**Figure 5:**
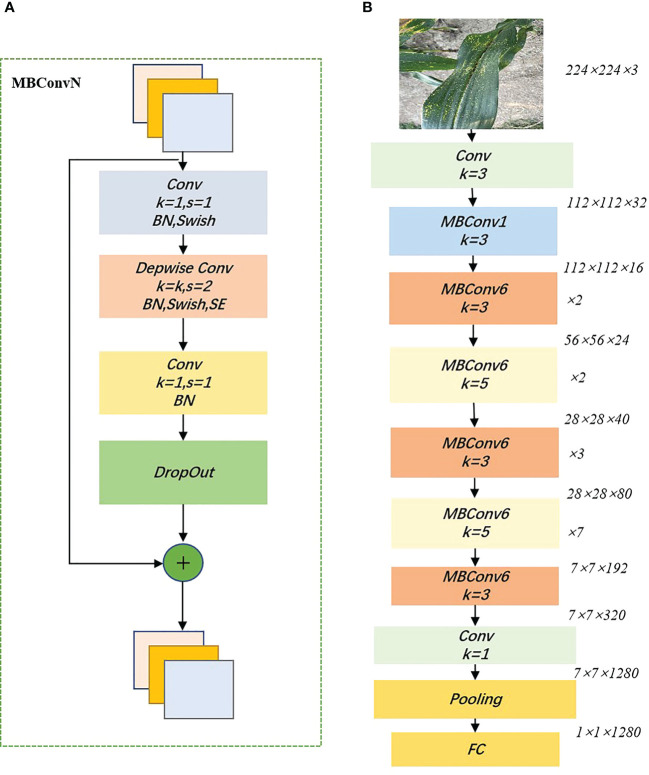
Building blocks of EfficientNet_b0. **(A)**:MBConv structure; **(B)** EfficientNet_b0 structure. swish indicates that the swish activation function is used, SE indicates that the Squeeze-and-Excitation module is added, and N indicates the multiplicity factor (i.e. The first convolutional layer in MBConv expands the channels of the input feature matrix by a factor of N), and BN denotes Batch Normalization.

EfficientNet is a lightweight convolutional neural network architecture and scaling method proposed by Google in 2020 that uniformly scales all dimensions of depth/width/resolution using a compound coefficient (Tan and Le, 2019). Unlike the traditional approach of scaling these factors arbitrarily, the EfficientNet scaling method uses a set of fixed scaling factors to uniformly scale the network width, depth, and separation rate. If the input image is larger, then the network needs more layers to increase the field of perception and more channels to capture finer-grained patterns on a larger image. The underlying network architecture of the model is designed using neural architecture search and the user can scale the model to suit their hardware resources. The core structure of EfficientNet is the mobile inverted bottleneck convolution (MBConv), which is obtained by searching through the neural network architecture, first convolving the input 1×1 point by point and varying the output channel dimension according to the expansion ratio. The depthwise convolution of k×k is then performed. influenced by the Squeeze-and-Excitation Network (SENet) (Hu et al., 2018), a compression and excitation operation is performed after the depth convolution, followed by a 1×1 point-by-point convolution ending reverting to the original channel dimension and performing a drop connect and an input skip connection, to improve the representational power of the network by making it possible to perform dynamic channel feature recalibration. Among other things, the MBConv module weighs network depth, width, and input image resolution by using simple and efficient composite coefficients. It allows the model to have a random depth, cutting short the time spent on training and inference. **Figure 5** shows the Building blocks of EfficientNet_b0.

#### 3.1.4 DenseNet

**Figure 6:**
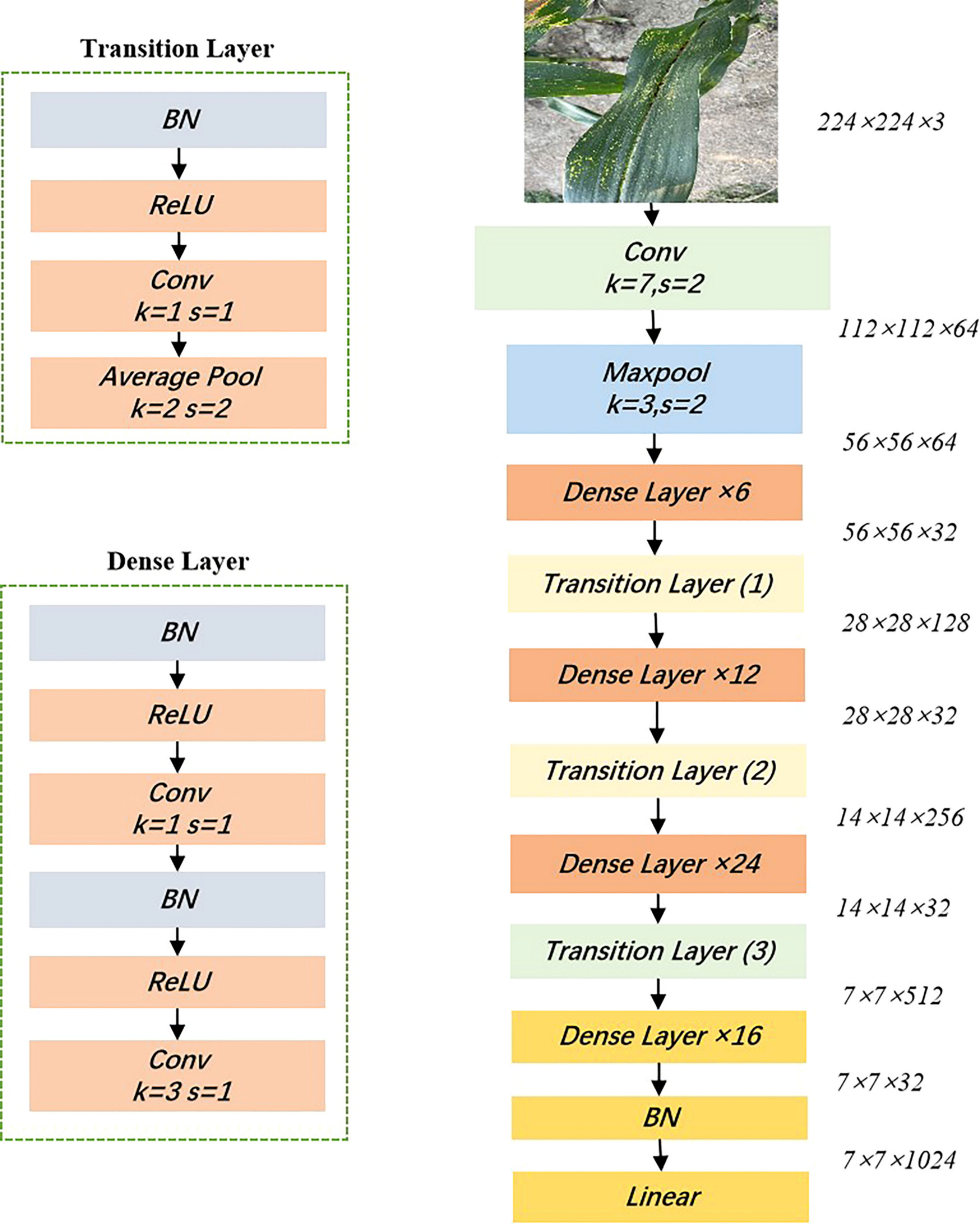
Structure of the DenseNet 121 model.

DenseNet is a convolutional neural network that exploits the potential of the network through feature reuse to produce condensed models that are easy to train and parameter efficient (Huang et al., 2017). Compared to ResNet, DenseNet proposes a more radical dense connectivity mechanism, where dense connectivity between layers is exploited through dense blocks to connect the feature maps learned by different layers, increasing the variability of inputs from subsequent layers and improving efficiency. For DenseNet, each layer is concatenated with all previous layers in the channel dimension and used as input to the next layer. CNNs generally go through Pooling or stride>1 convolution to reduce the size of the feature map, whereas the densely connected approach of DenseNet requires the feature map size to be consistent. To solve this problem, DenseNet uses the structure of DenseBlock+Transition, as shown in **Figure 6**, where DenseBlock is a module containing many Dense layers, each layer has the same feature map size, and the layers are densely connected. The advantages of DenseNet are: (1) Due to the dense connection, DenseNet improves the backpropagation of the gradient, making the network easier to train. Since each layer can go straight to the final error signal, implicit deep supervision is achieved; (2) the parameters are small and computationally efficient, and feature reuse is achieved since DenseNet is short-circuited by fusing features to achieve short-circuited connections. In this study, DenseNet121 was chosen for training and testing.

### 3.2 CAM-based method for locating maize leaf spots

Class Activation Mapping (CAM) is a class response map generated from a classification network. It can roughly localize to discriminative object regions in an image based on image-level annotation information (Zhang et al., 2022). It reveals the distribution of the CNN’s contribution to the prediction output, with higher scores indicating a higher response and greater contribution to the network from the corresponding region of the original image. It has the following 3 advantages: (1) it helps to understand and analyze how neural networks work and the decision-making process, which in turn helps us to better select and design the network, for example, for classification networks, we need high prediction accuracy on the one hand, and on the other hand, we also need the network to extract the features we want to obtain; (2) using the visualization of the network response can guide the network to learn better, for example, we can use the information reflected by CAM to enhance the data by cropping, etc.; (3) using CAM as a basis for weakly supervised semantic segmentation or weakly supervised localization. Because CAM can cover the target object, it is possible to use only classification annotation to complete the semantic segmentation or target detection task, which greatly reduces the workload of annotation, but this is more demanding on CAM. In general, the classification network will only extract the most discriminative features.

**Figure 7:**
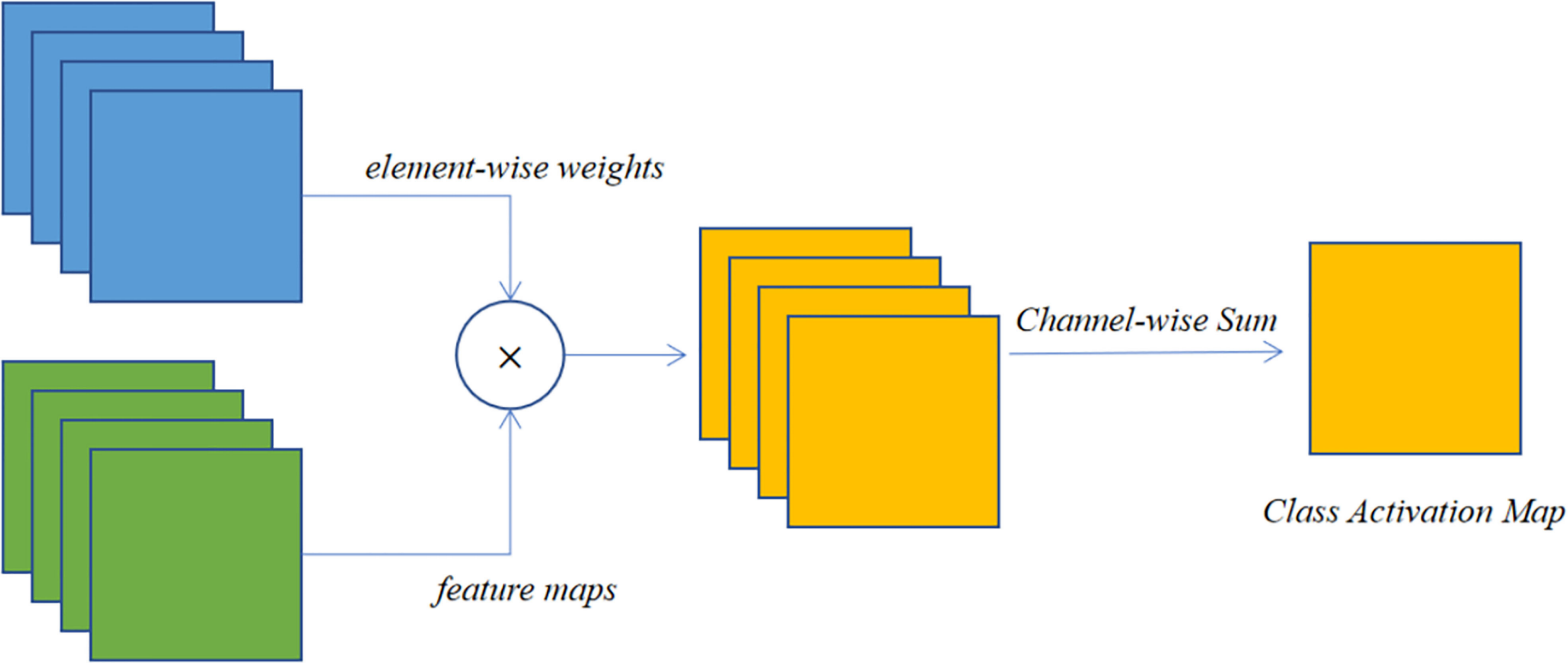
The process of class activation mapping methods.


On the other hand, the CAM method can project the features extracted by the network onto the input image, so the image can be examined to see how the classifier is behaving. If the classifier behaves correctly, these features may represent the location of the crop disease, however, if the classifier is extracting features that are not related to the disease, then problems with the classifier can be identified in time. This type of approach is important from a practical point of view as it projects the features extracted from the network onto the input image and therefore allows the way in which the classifier behaves to be understood by examining the image. If the classifier behaves correctly, then these parts may be representative of symptoms or features of the disease. This is the case if the classifier uses the background or another feature that is not related to the plant disease as the basis for classification. The extraction of CAM generally occurs at the convolutional layer, and in particular at the last layer of convolution. **Figure 7** illustrates the general process of these methods. It is generated by the interaction between the convolutional layer, the global average pooling layer, and the CNN classification layer (Jiang et al., 2021b). The CAM for a given category a is defined as $M_{a}$ , and each spatial element can be represented by equation (1).

(1) $$M_{a}(x,y)=\sum_{l=1}^{L}\omega_{l}^{a}f_{l}(x,y)$$

where $f_{l}(x,y)$ denotes the activation of unit l in the final convolution layer at spatial location (x,y) of a given feature map, and $\omega_{l}^{a}$ denotes the weight of the unit l associated with class a . Thus, $M_{a}(x,y)$ activation at spatial position (x,y) plays a key role, which in turn classifies the image into class a , and by simply upsampling the CAM to the same size as the input image, the image regions most relevant to a particular category can be identified.

Similar to anchor-base and anchor-free in the field of target detection, CAM is also divided into gradient-based and gradient-free, both of which extract target feature layers and perform weighted fusion to obtain CAM. The difference lies in the selection of the fusion weights between the feature layers, with gradient-based using gradients to obtain the weights, while gradient-free does not require gradient information. This section presents state-of-the-art CAM methods based on both types.

#### 3.2.1 LayerCAM

Traditional CAM methods, such as GradCAM (Selvaraju et al., 2017) and GradCAM++, can only generate class activation maps from deep layers of the convolutional neural network. Due to the small spatial resolution of the final convolutional layer, the class activation mapping map can usually only locate a coarse region of the target. LayerCAM (Jiang et al., 2021b), on the other hand, can generate reliable class activation mapping maps for different convolutional layers of a CNN by generating individual weights for each spatial location in the feature map using backward class-specific gradients. The strengths of the class activation mapping maps of the different convolutional layers are complemented to generate more accurate and complete class-specific target regions. Furthermore, LayerCAM can be applied directly to CNN-based deep learning image classifiers without the need to modify the network architecture and back-propagation methods. The class activation mapping $M^{c}$ generated by LayerCAM is shown in Equations (2), and (3).


(2) $$y^{c}=f^{c}(I,\theta )$$

(3) $$M^{c}=ReLU(\sum_{k}\left(relu(\frac{\partial y^{c}}{\partial A_{ij}^{k}})\cdot A_{ij}^{k})\right)$$

Where c is the target category, f denotes the image classifier, θ denotes the parameters of the classifier, and I denotes the input image. $y^{c}$ denotes the prediction score for obtaining the target category c , A is the output feature map of the final convolutional layer in the CNN, and $A_{ij}^{k}$ denotes the value of the spatial location ( i,j ) in the first k feature map in A .

#### 3.2.2 ScoreCAM

ScoreCAM is a new CAM-based gradient-free visual interpretation method (Wang et al., 2020). Unlike previous CAM-based methods, ScoreCAM obtains the weights of each activation mapping by its positive transfer score on the target class, thus getting rid of the dependence on gradients, and the final result is obtained by a linear combination of weights and activation mappings. It bridges the gap between perturbation-based and CAM-based methods and dictates the weights of the activation maps in an intuitive and understandable way. Using the notation in Section 3.2.1, the class activation mapping map $L_{Score-CAM}^{c}$ generated by Score-CAM can be defined according to Equation (4), assuming a convolutional layer l in the image classifier f , given an interest class c , as


(4) LScore−CAMc=ReLU(∑kC(Alk)Alk)

where C(·) denotes the Channel-wise Increase of Confidence(CIC) score of the class activation graph $A_{l}^{k}$ .

#### 3.2.3 AblationCAM

AblationCAM is a gradient-free visual interpretation method for deep convolutional neural networks that avoids the use of gradients while producing high-quality class-distinct localization maps (Ramaswamy, 2020). AblationCAM can be a good solution to the problem of not providing convincing interpretations and highlighting relatively small incomplete regions of objects in an image due to gradient saturation. The class activation mapping map $L_{Ablation-CAM}^{c}$ generated by AblationCAM is shown in equation (5):


(5) $$L_{Ablation-CAM}^{c}=RELU\left(\sum_{k}\frac{y^{c}-y_{k}^{c}}{y^{c}}A_{k}\right)$$

Where $y^{c}$ is the class activation score obtained by the model through forward pass, $y_{k}^{c}$ is the score of class c obtained after the kth channel of the feature map is all set to 0.

#### 3.2.4 XGradCAM

How weights are determined has always been a key issue in CAM visualization, and different definitions of weights produce different CAM methods. In order to provide a basis for the solution of the weights, XGradCAM (Fu et al., 2020) introduces two axioms in the derivation process: Sensitivity and Conservation; the role of Sensitivity is that when a feature mapping is set to zero, the more significant the decrease in a score, the more important the feature mapping should be. Conservation was introduced to ensure that the category scores are dominated by feature mapping and not by some other uncontrollable factor. The equations for the $M_{c}^{XGrad-CAM}$ of the class activation mapping generated by XGrad-CAM are shown in (6), and (7).


(6) $$\alpha_{c}^{k}=\sum_{x,y}\left(\frac{F^{lk}(x,y)}{\sum_{x,y}F^{lk}(x,y)}\cdot \frac{\partial S_{c}(F^{l})}{\partial F^{lk}(x,y)}\right)$$

(7) $$M_{c}^{XGrad-CAM}(x,y)=\sum_{k=1}^{K}\left(\alpha_{c}^{k}F^{lk}(x,y)\right)$$

Where $F^{lk}(x,y)$ denotes the xth row and yth column response of the kth feature mapping in the lth layer of the network and $S_{c}\left(F^{l}\right)$ is the c class score predicted by the CNN. A ReLU correction to $M_{c}^{XGrad-CAM}$ is also required in order to highlight those regions that play a positive role in the classification results. In addition, the corrected class activation mappings need to be upsampled to the size of the input image as the deeper feature mapping size is usually smaller than the size of the input image.

In summary, this study will compare the effectiveness of each of the above four CAM methods in locating areas infected with maize leaf spots. The differences between the different methods are shown in **Table 2**.

**Table 2:** 4 state-of-the-art CAM methods.

Type	Method	Advantages
gradient-based	LayerCAM	Using element-level weights to generate higher-quality class activation maps
gradient-free	ScoreCAM	Infiltrate the image by scaling activation and measure how the output drops
gradient-free	AblationCAM	Zeroing out the activation and measuring the drop in output
gradient-based	XGradCAM	Scaling gradients by normalizing activation

### 3.3 Performance evaluation indicators

In order to present statistics on accurate and incorrect image recognition, this study uses F1 scores, FLOPs (floating-point operations), and FPS (frames per second) as evaluation metrics, with F1 scores representing the summed average of Precision and Recall (Barstugan et al., 2020). The F1 score represents the summed average of Precision and Recall, where Precision represents the percentage of predicted positive values that are actually positive, and Recall represents the percentage of predicted positive values that are actually positive. FLOPs represent the amount of computation used to measure the complexity of the model, and FPS represents how many frames per second the network can process. The equations for calculating the metrics are shown in (8), (9), and (10).


(8) $$\text{P}(Precision)=\frac{\text{TP}}{\text{TP}+\text{FP}}$$

(9) $$\text{R}(\text{Recall})=\frac{\text{TP}}{\text{TP}+\text{FN}}$$

(10) $$\text{F}1\text{ Score}=\frac{2*\text{P}*\text{R}}{\text{P}+\text{R}}$$

Where TP(True Positive) indicates the number of positive instances predicted correctly, FN(False Negative) is the number of positive instances classified incorrectly; FP(False Positive) is the number of negative instances classified as a positive category and TN(True Negative) is the number of negative instances that have been accurately classified.

**Figure 8:**
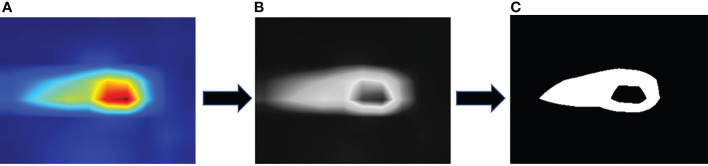
Image processing process: **(A)** Heat map, **(B)** Grayscale map, **(C)** Binary map.


In evaluating the performance of the CAM algorithm for locating the location of image spots, we first perform class prediction on the disease image, then generate a class activation mapping map as shown in **Figure 8** and convert the generated heat map to a grey-scale map. Here we design a gradient experiment to convert the greyscale map into a binary map by setting the operational thresholds used to generate image boundaries from the score map to 50%, 60%, and 70% of the maximum pixel value in the greyscale map, respectively, and keeping the pixels with values above the threshold according to the thresholds we set. The aim was to extract closer to the actual contours and locations of the lesions from the class activation mapping map. At the same time, we manually labeled the real contours of the lesions and calculated the IoU (Intersection over Union) of the two, with the equation shown in (11).

(11) $$\text{IoU}=\frac{Area\;of\;Overlap}{Area\;of\;Union}$$

### 3.4 Transfer learning

Transfer learning is a machine learning method (Tan et al., 2018) that works by taking knowledge from one domain (the source domain) and transferring it to the target domain, enabling the target domain to achieve better learning results (Özkaya et al., 2020). Usually, when the source domain has an adequate amount of data and the target domain has a small amount of data, this situation lends itself to the use of migration learning. By using migration learning, the model can have better initialization performance and accelerate the learning and optimization of the network during the training process. ImageNet is a large visualization database for visual object recognition software research (Krizhevsky et al., 2017), which contains about 1.2 million images and 1000 categories. Many researchers have used this dataset as a source domain for migration learning (Diaz-Romero et al., 2021; Relekar and Shanmugam, 2021; Chen et al., 2022). This migration learning strategy is a frequent training method when training CNNs using image data. The migration learning approach requires only exponentially fewer data to learn specific features of a custom class. The reduced amount of data required significantly reduces training time and data collection, making CNNs easier to use for everyday tasks.

## 4 Experimental analysis and discussion

All processes used in this study were based on Python 3.7 under Linux and the PyTorch deep learning framework. The server CPU was an Intel(R) Xeon(R) CPU E5-2678 v3, 64 GB of RAM, and included two Nvidia RTX 2080 graphics processing units (GPUs). This experiment is divided into 2 parts: (1) the basic models of maize leaf spot classification were obtained by training MobileNetV3_small, ShuffleNetV2, EfficientNet_b0, and DenseNet121 networks with enhanced training sets; (2) the above 4 networks were combined with LayerCAM. ScoreCAM, AblationCAM, and XGradCAM, respectively, to obtain the best-performing maize leaf spot classifier. Among them, Score-CAM and Ablation-CAM need a large number of forward passes, and we set the batch size of forward pass to 128. The network parameters of the classification models are shown in **Table 3**. In addition, all models were trained on a data-enhanced training set, and the weights of the models were pre-trained on ImageNet. **Table 4** shows the hyperparameters of the training process.

**Table 3:** Network parameters of the classification model.

Network parameters	DenseNet	EfficientNet	MobileNetV3	ShuffleNetV2
Total params	7,978,856	5,288,548	2,542,856	2,278,604
Forward/backward pass size(MB)	172.18	173.65	34.61	47.94
Params size(MB)	30.44	20.17	9.70	8.69
Estimated Total Size(MB)	203.19	194.40	44.88	57.20

**Table 4:** Hyperparameters in the training process.

hyper-parameters	Value
Epochs	300
Batch size	32
Optimizer	SGD
Momentum	0.9
Weight decay	0
Learning rate decay	cos
Learning rate	2e-5~2e-3

### 4.1 Performance comparison of various lightweight network classifications

**Figure 9:**
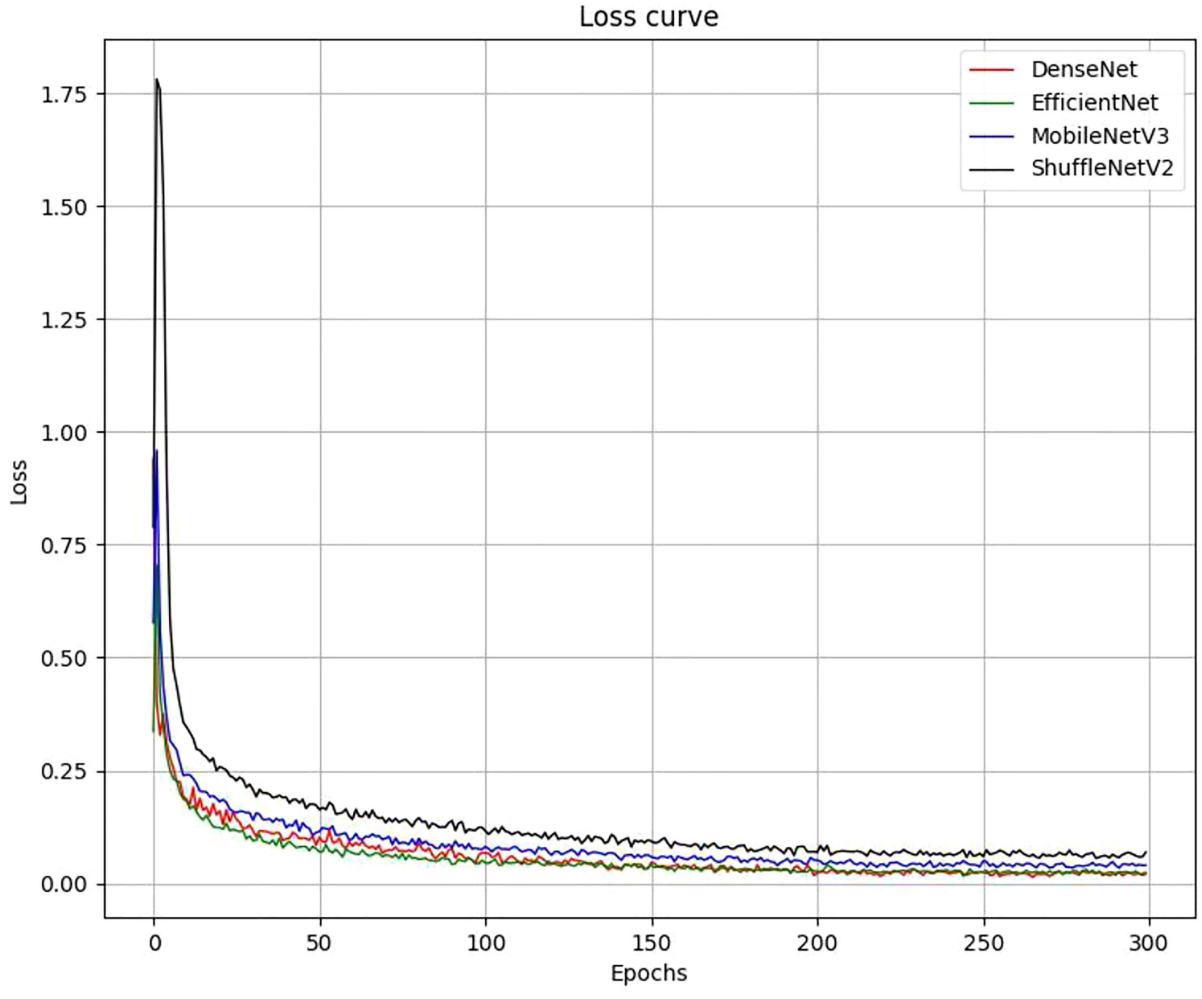
Loss curves for each network during training.

As shown in **Figure 9**, the losses of the 4 networks were recorded after each training period, and after 300 iterative training cycles, the training loss values of all networks stabilized, indicating that all 4 models had converged. After the training was completed, we used 5-fold cross-validation to evaluate the classification effectiveness of the 4 networks. **Table 5** shows that there is no direct relationship between the model parameters, computational effort, and the final classification performance, with the F1 score of EfficientNet_b0 being higher than that of the other three models. In terms of recognition speed, MobileNetV3 is the fastest of the four lightweight networks.

**Table 5:** Performance evaluation of different network architectures.

Networks	FLOPs	Total params	R	P	F1 Score	FPS
DenseNet	5.794G	7.979M	95.80%	96.33%	95.983%	62.89
EfficientNet	830.290M	5.289M	95.58%	96.49%	96.049%	132.65
MobileNetV3	124.956M	2.543M	94.97%	95.33%	95.072%	207.41
ShuffleNetV2	305.418M	2.279M	95.56%	95.57%	95.579%	183.57

**Figure 10:**
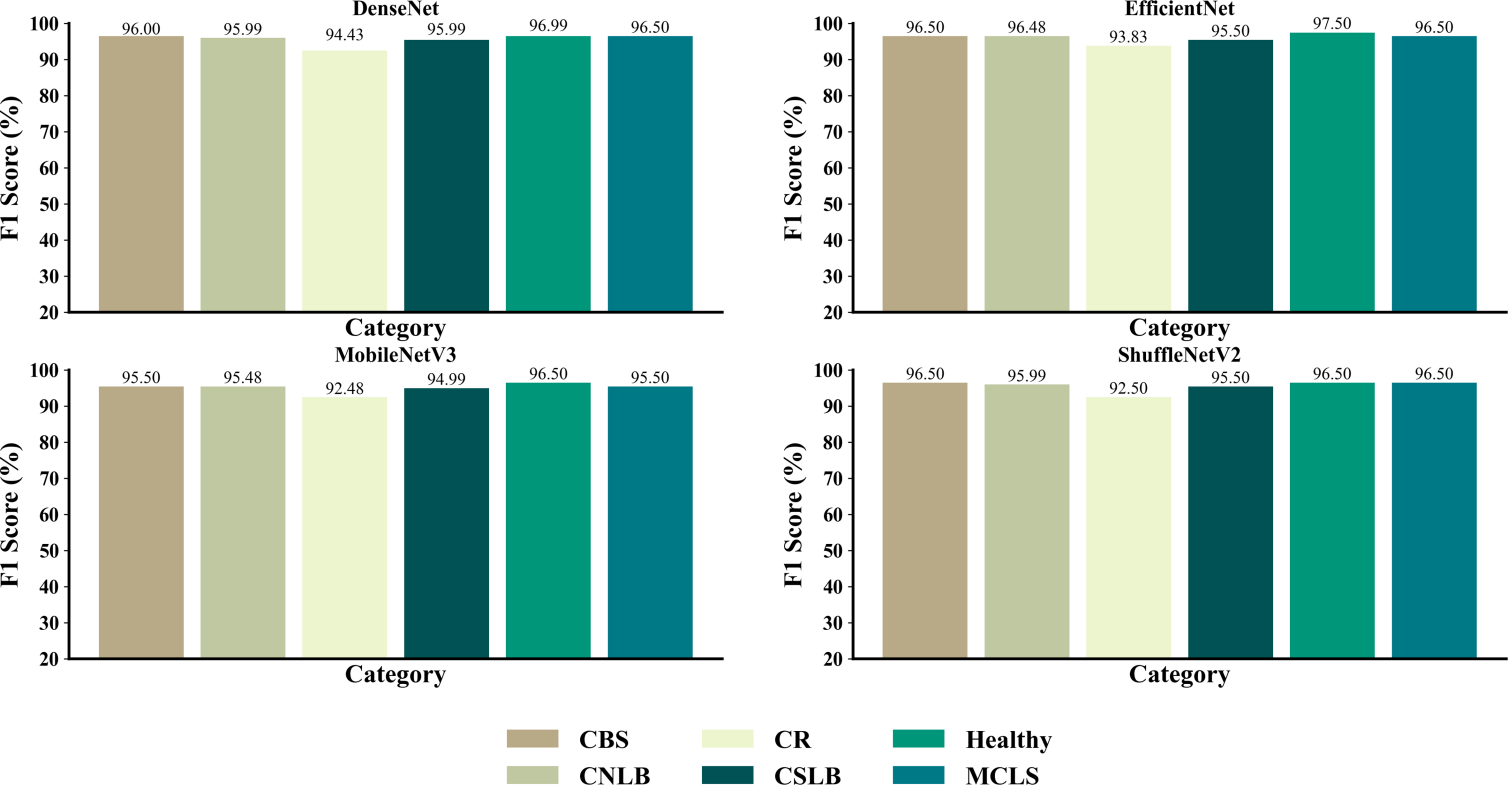
F1 scores for each type of disease for different networks.

**Figure 11:**
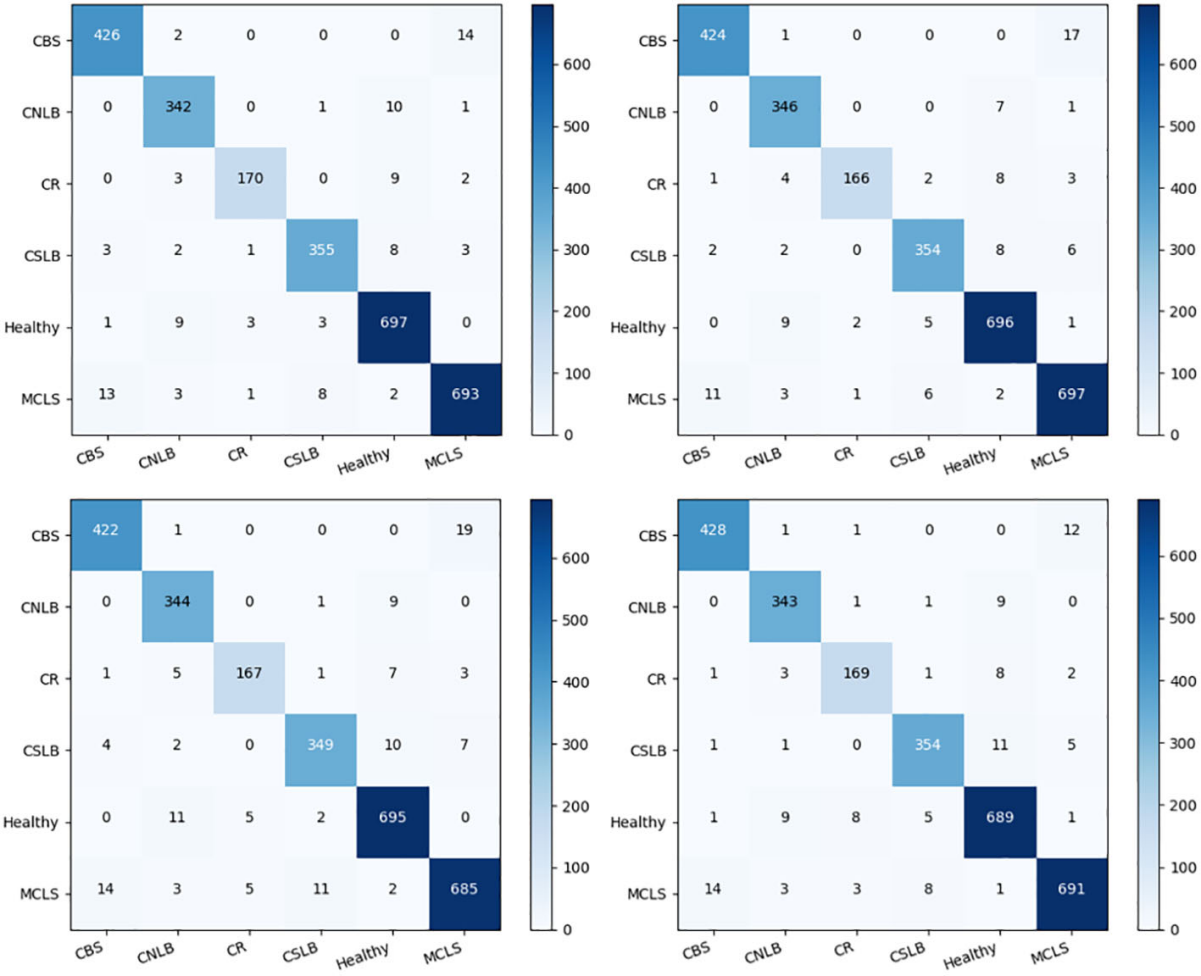
Confusion matrix for the predicted results of each model.

As shown in **Figure 10**, the four networks identified the different types of maize leaf spots, with all four models achieving good F1 scores for all types of maize leaf spots. **Figure 11** shows the confusion matrix of the predicted results for each model. There were instances where each model confused MCLS with CBS, as the two diseases were extremely similar in appearance, with only minor differences in some locations. There was also a misclassification of healthy classes into CNLB, possibly because some healthy classes had tiny spots on the leaves, but not enough to be considered non-healthy. On the other hand, complex field backgrounds and different light intensities can also affect feature extraction from disease images, leading to incorrect individual classification.

### 4.2 Comparison of the effectiveness of CAM-based localization of infected areas

**Figure 12:**
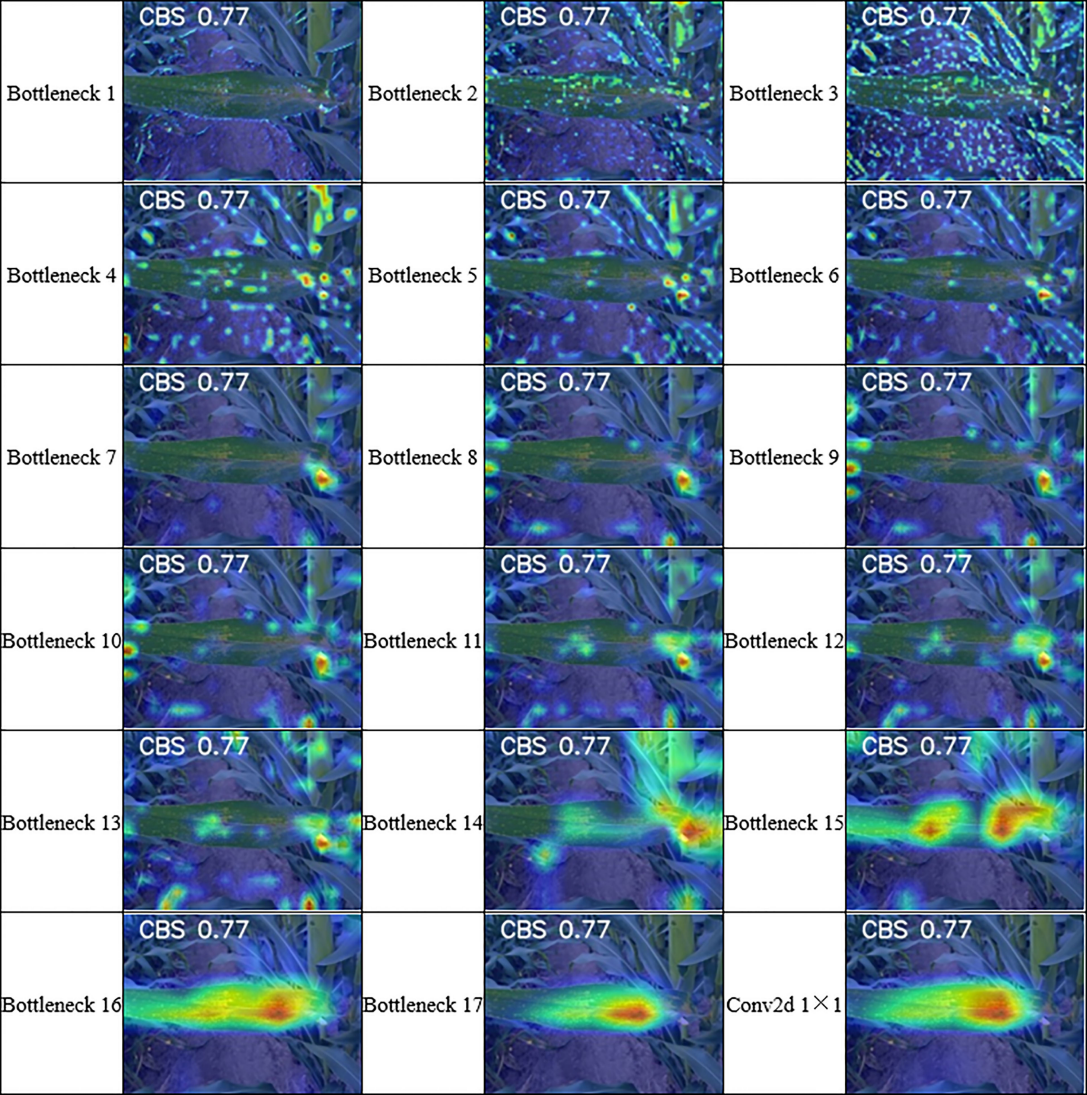
Presentation of class activation mapping based on MobileNetV2 per layer extraction.

To further understand the details about the deep learning classification mechanism and whether the network extracted the features we wanted to obtain, we also wanted to know the deviation of the disease spot locations extracted by CAM from the actual spot locations. We extracted the disease features we wanted to obtain in four lightweight networks using the 2 CAM methods mentioned in 3.2 and evaluated the effect of class activation mapping. As each layer in the model extracts different features, in general, deeper representations in the CNN capture the higher-level visual structure and the convolutional features retain spatial information, but this information is lost in the fully connected layers. As shown in **Figure 12**, we used the example of GradCAM extracting features from CBS, and the deep learning classifier did not extract the features we wanted when using the network layer before the 16th Bottleneck residual block as the target layer. So in this study, we randomly selected 453 disease images from the validation set for testing the localization of infected areas in maize leaves. These included 88 CBS, 83 CNLB, 96 CR, 95 CSLB, and 76 MCLS. we selected the last convolutional layer of each network as the target layer for class activation mapping and evaluated it, and then set the boundary thresholds to 50%,60%, and 70% of the maximum pixel value in the grey-scale map according to Section 3.3, respectively, to analyze the performance of these CAM methods were analyzed for their effectiveness in locating infected areas of maize leaf diseases under different thresholds. We manually annotated the images in the test set and evaluated the localization effect by calculating the boundary contours of the class activation mapping map and the IoU of the manually annotated contours.

**Figure 13:**
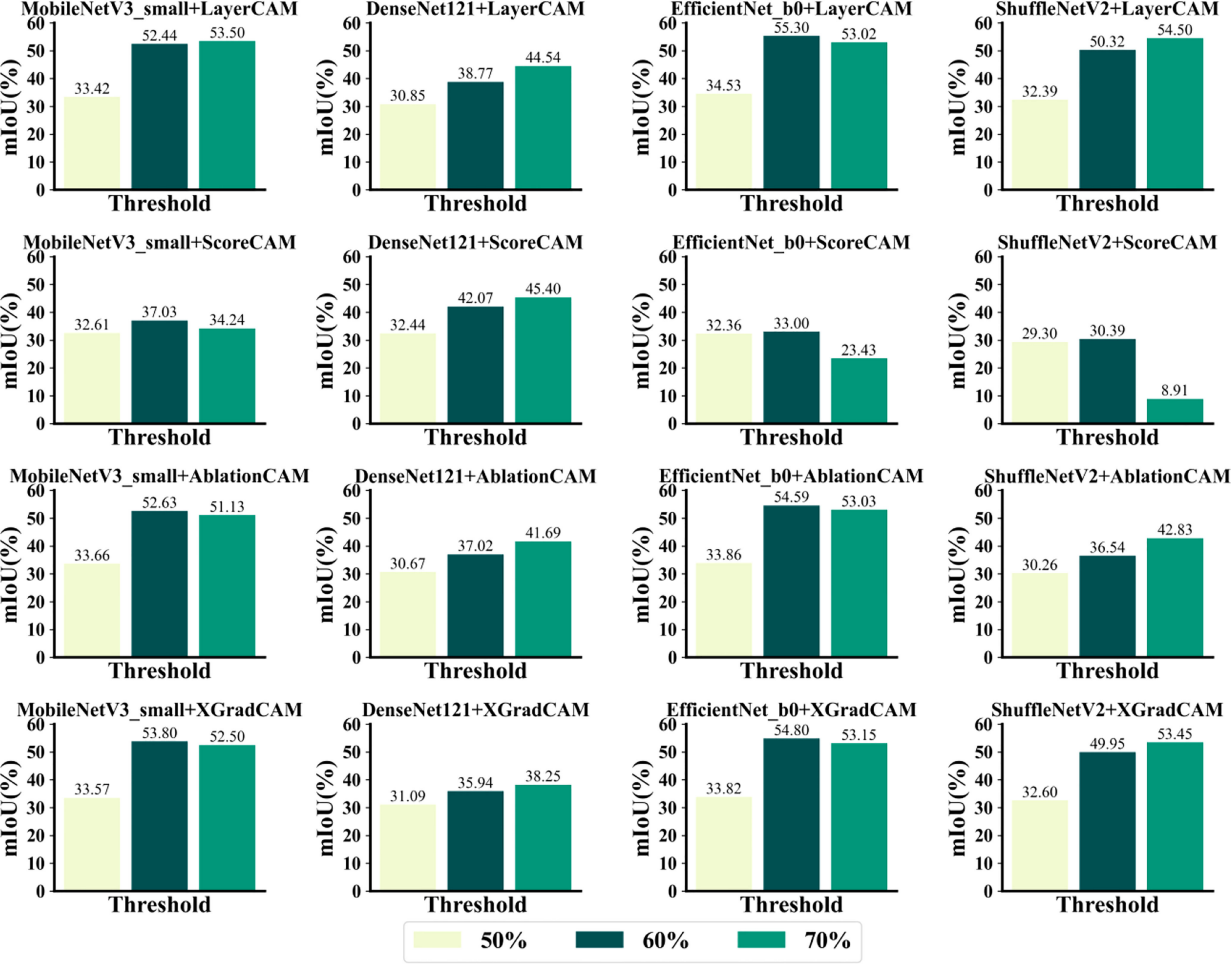
Effectiveness of each CAM method in localizing the infected area.

As shown in **Figure 13**, the localization results were generally lower than 60% and 70% when the threshold value was set to 50%. When the threshold value was set to 50%, the combination of EfficientNet_b0+LayerCAM could achieve the highest mIoU of 55.302% for the infected area of maize leaves. When the threshold was set to 70%, the combination of ShuffleNetV2+ScoreCAM was the least effective, and ScoreCAM was the least effective of the four CAM methods in locating infected areas. **Table 6** compares the best spot localization accuracy that each CAM method can achieve, from which it can be seen that the combination of EfficientNet_b0 and LayerCAM, AblationCAM, and XGradCAM all achieve a mIoU of more than 54% when relying only on image-level annotation. On the other hand, the four networks were generally better at locating CBS and CSLB than the other three infestations. The localization effect on the infected zone of CNLB was the worst among the five diseases. On the other hand, ScoreCAM and AblationCAM took relatively longer because AblationCAM had to traverse each feature map to ablate it and check for changes in the class activation scores and check for decreases in the corresponding class activation scores. So it takes longer to map the activation of the features. And DenseNet121 is more computationally intensive than the other 3 networks, so it takes longer to generate the class activation mapping maps. layerCAM and XGradCAM require a single backpropagation to generate the class activation mapping maps, which can be significantly reduced if they are properly multi-processed.

**Table 6:** Optimal mIoU achieved by different CAM methods.

s									
LayerCAM	EfficientNet	60%	61.68%	39.7%	54.81%	63.38%	56.94%	55.302%	01:12
ScoreCAM	DenseNet	70%	54.54%	28.69%	44.88%	49.07%	49.82%	45.4%	41:04
AblationCAM	EfficientNet	60%	61.81%	38.69%	55.86%	62.56%	54.04%	54.592%	26:22
XGradCAM	EfficientNet	60%	62.13%	38.6%	55.54%	63%	54.71%	54.796%	01:12

**Figure 14:**
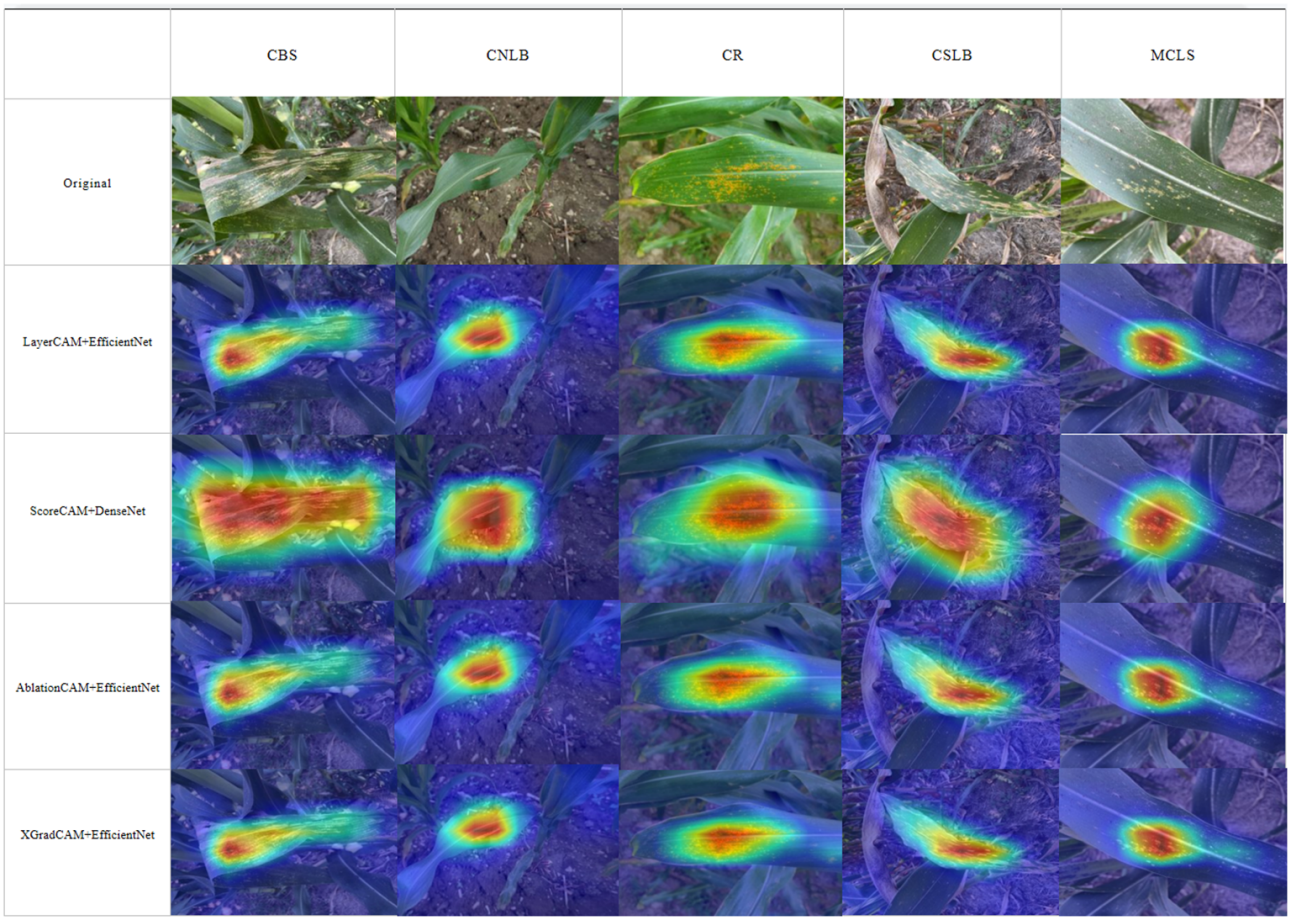
Class activation mapping.

**Figure 14** shows a visual sample of the combination of the four CAM methods with the best localization performance. It can be seen that ScoreCAM localizes a much larger range, including areas unrelated to the lesion, which is responsible for ScoreCAM’s poorer localization performance than the other three CAMs. As the weights are derived from the CIC scores corresponding to the target class activation maps, ScoreCAM is free from the dependence on gradients. Since the weight of each activation map is represented by its score for the target class, each target object predicted by the model with a high confidence score can be highlighted independently. Thus, all evidence associated with the target class can be responded to and assembled by linear combination. On the other hand, the four methods have relatively low localization refinement for CNLB, which may be due to the fact that the CNLB images in the dataset are not as rich, thus leading to the weakly supervised semantic segmentation based on the CAM method not being able to generalize the learned features well. In summary, it is feasible to achieve localization of maize leaf spots by CAM-based weakly supervised semantic segmentation.

In comparison to the currently available research results, Md. Ashraful Haque et al. achieved an overall classification accuracy of 95.99% and an average recall of 95.96% on a dataset of 4 types and 5939 maize disease images through the Inception-v3 network framework (Haque et al., 2022). Although this study also achieved good performance, it was unable to localize infected areas and the model was not very interpretable. 91.83% accuracy was achieved by Sun et al. using a CNN model for the identification of maize maculate spots, but the drawback of this was the small variety and number of samples and the reliance on more detailed annotation information (Sun et al., 2020).

## 5 Conclusion

Based on the constructed dataset of maize leaf spot disease in the field environment, this study proposes a maize leaf spot disease recognition model that combines lightweight deep learning classifiers with visualization techniques for the identification and localization of leaf spot disease in maize in the field. We selected four lightweight networks as backbone networks, used pre-trained models on the ImageNet dataset to initialize the weights of deep learning classifiers, and combined them with four state-of-the-art interpretable AI algorithms based on CAM to evaluate the effectiveness of weakly-supervised learning for locating infected zones. The experimental results demonstrate that a lightweight CNN architecture based on weakly supervised learning is able to learn and predict the location of infected zones in maize leaf disease images, despite being trained from complex field scenes with only image-level annotations. While the approximate location of disease spots can be predicted fairly reliably by weakly supervised learning, the accuracy of the predictions is not good enough, due to the network’s tendency to focus on unique regions. We believe that weakly supervised learning has greater potential for exploitation in the plant pest and disease domain, as it effectively addresses the over-reliance on manual labeling in previous related studies. In contrast to traditional fully supervised learning methods, weakly supervised learning requires the manipulation of training data with weak labels to learn the target model, thus alleviating the cost of annotating training samples. It can also facilitate the learning process when the fine-grained annotation is very time-consuming.

On the other hand, although the approximate location of the disease can be located by CAM methods, it cannot achieve the same accuracy as in the target detection task. In classification tasks, the model tends to base its judgment on the most salient and discriminative regions of the object, so during training, the classification model will increasingly favor these regions, so that the classification score of proposals containing these local regions will be higher and higher, and therefore the classification score of proposals covering only these local regions will naturally be the highest. So this is why weakly supervised semantic segmentation often does not cover the entirety of the target object, as it only shows the local optimal solution. Also for some users, it is necessary to quantify the severity of the disease in the maize leaves. Whether this information can be extracted from the heat map remains to be investigated.

## Data availability statement

The raw data supporting the conclusions of this article will be made available by the authors, without undue reservation.

## Author contributions

YS wrote the first draft of the manuscript. XZ and WH contributed to the conception and design of the study. GX and DX organized the database. YY carried out statistical analysis. ZR, ZX, LS, YZ, and ZL wrote part of the manuscript. All authors contributed to the revision of the manuscript, read and approved the submitted version
